# Sweetened Beverage Tax Implementation and Change in Body Mass Index Among Children in Seattle

**DOI:** 10.1001/jamanetworkopen.2024.13644

**Published:** 2024-05-29

**Authors:** Jessica C. Jones-Smith, Melissa A. Knox, Suman Chakrabarti, Jamie Wallace, Lina Walkinshaw, Stephen J. Mooney, Jessica Godwin, David E. Arterburn, Joanna Eavey, Nadine Chan, Brian E. Saelens

**Affiliations:** 1 Department of Health Systems and Population Health, University of Washington, Seattle; 2 Department of Epidemiology, University of Washington, Seattle; 3 Department of Economics, University of Washington, Seattle; 4 Nutrition, Diets and Health Unit, International Food Policy Research Institute, New Delhi, India; 5 Center for Studies in Demography and Ecology, University of Washington, Seattle; 6 Kaiser Permanente Washington Health Research Institute, Seattle; 7 Public Health—Seattle and King County, Seattle, Washington; 8 Department of Pediatrics, University of Washington, Seattle; 9 Seattle Children’s Research Institute, Seattle, Washington

## Abstract

**Importance:**

Sweetened beverage taxes have been associated with reduced purchasing of taxed beverages. However, few studies have assessed the association between sweetened beverage taxes and health outcomes.

**Objective:**

To evaluate the association between the Seattle sweetened beverage tax and change in body mass index (BMI) among children.

**Design, Setting, and Participants:**

In this longitudinal cohort study, anthropometric data were obtained from electronic medical records of 2 health care systems (Kaiser Permanente Washington [KP] and Seattle Children’s Hospital Odessa Brown Children’s Clinic [OBCC]). Children were included in the study if they were aged 2 to 18 years (between January 1, 2014, and December 31, 2019); had at least 1 weight measurement every year between 2015 and 2019; lived in Seattle or in urban areas of 3 surrounding counties (King, Pierce, and Snohomish); had not moved between taxed (Seattle) and nontaxed areas; received primary health care from KP or OBCC; did not have a recent history of cancer, bariatric surgery, or pregnancy; and had biologically plausible height and BMI (calculated as weight in kilograms divided by height in meters squared). Data analysis was conducted between August 5, 2022, and March 4, 2024.

**Exposure:**

Seattle sweetened beverage tax (1.75 cents per ounce on sweetened beverages), implemented on January 1, 2018.

**Main Outcomes and Measures:**

The primary outcome was BMIp95 (BMI expressed as a percentage of the 95th percentile; a newly recommended metric for assessing BMI change) of the reference population for age and sex, using the Centers for Disease Control and Prevention growth charts. In the primary (synthetic difference-in-differences [SDID]) model used, a comparison sample was created by reweighting the comparison sample to optimize on matching to pretax trends in outcome among 6313 children in Seattle. Secondary models were within-person change models using 1 pretax measurement and 1 posttax measurement in 22 779 children and fine stratification weights to balance baseline individual and neighborhood-level confounders.

**Results:**

The primary SDID analysis included 6313 children (3041 female [48%] and 3272 male [52%]). More than a third of children (2383 [38%]) were aged 2 to 5 years); their mean (SE) age was 7.7 (0.6) years. With regard to race and ethnicity, 789 children (13%) were Asian, 631 (10%) were Black, 649 (10%) were Hispanic, and 3158 (50%) were White. The primary model results suggested that the Seattle tax was associated with a larger decrease in BMIp95 for children living in Seattle compared with those living in the comparison area (SDID: −0.90 percentage points [95% CI, −1.20 to −0.60]; *P* < .001). Results from secondary models were similar.

**Conclusions and Relevance:**

The findings of this cohort study suggest that the Seattle sweetened beverage tax was associated with a modest decrease in BMIp95 among children living in Seattle compared with children living in nearby nontaxed areas who were receiving care within the same health care systems. Taken together with existing studies in the US, these results suggest that sweetened beverage taxes may be an effective policy for improving children’s BMI. Future research should test this association using longitudinal data in other US cities with sweetened beverage taxes.

## Key Points

**Question:**

Is the implementation of a sweetened beverage tax in Seattle, Washington, associated with a change in body mass index (BMI) among children living in Seattle?

**Findings:**

In this cohort study of 6313 children living in Seattle or a nearby comparison area, a statistically significant reduction in BMI was observed for children in Seattle after the implementation of a sweetened beverage tax compared with well-matched children living in nontaxed comparison areas.

**Meaning:**

These results suggest that the sweetened beverage tax in Seattle may be associated with a small but reasonable reduction in BMI among children living within the Seattle city limits.

## Teaser

This cohort study examines the association between the Seattle sweetened beverage tax and change in body mass index (BMI) among children living in Seattle compared with those living in nearby nontaxed areas.

## Introduction

To date, 7 US cities have implemented excise taxes on sweetened beverages. These taxes were pursued with the goal of improving population health by disincentivizing intake of sugar-sweetened beverages, the single largest contributor to added sugar intake in the US.^1^ A secondary goal of these taxes has been to increase revenues, often targeted toward other public health programs.^2^

Sweetened beverage taxes have generally been shown to increase prices of taxed beverages and decrease purchasing of taxed beverages.^3,4,5,6,7,8,9,10^ Studies have reported a net decline in grams of added sugar purchased, suggesting a potential for net reductions in calories consumed.^11^ At the same time, the existing literature suggests the association of sweetened beverage taxes with self-reported sugary beverage consumption has tended to be null.^10^ However, dietary consumption is difficult to measure, and small studies with inadequate power paired with small expected effect sizes can lead to null findings.^12^ Therefore, it remains important to assess whether these taxes have had detectable associations with health outcomes.

There are several reasons to believe that sweetened beverage taxes may be associated with children’s body mass index (BMI; calculated as weight in kilograms divided by height in meters squared). First, US residents tend to consume more sweetened beverages during adolescence than during adulthood, so taxes may more substantially affect their dietary intake.^13^ Second, adults with children may change the beverages they provide their children in response to either price changes or health-signaling effects of taxes on goods such as sweetened beverages. Children may change their consumption habits as a result of either mechanism. Finally, the prevention of increases in BMI among children might be more physiologically tenable compared with incurring weight loss among adults.^14^

Three previous studies have examined associations between sweetened beverage taxes and children’s weight status. The first study examined BMI among children in Mauritius, and no detectable association between the Mauritius sugar-sweetened beverage tax and BMI was observed among boys or girls.^15^ In the second study, investigators found that pass-through of the Mexico sweetened beverage tax was associated with a reduction in obesity prevalence among adolescent girls, but not boys.^16^ The third study examined sweetened beverage taxes in 3 cities (Philadelphia, Pennsylvania; San Francisco, California; and Oakland, California), and beverage taxes were associated with a decrease in average BMI among all children, but with larger effects among girls.^17^ Each of these studies used repeated cross-sectional samples.

In this study, we built on the aforementioned recent work to identify the population health consequences of sweetened beverage taxes. We used longitudinal, measured BMI data from a large sample of children in urban areas, with anthropometrics from electronic health records to assess whether exposure to the Seattle sweetened beverage tax was associated with change in BMI from before to after the tax was implemented. We hypothesized that the tax would be associated with lower gains in BMI.

## Methods

This cohort study was approved by the University of Washington Institutional Review Board (IRB), which determined that the activities conducted by the research team were nonhuman participant research; therefore, informed consent was not required. The IRBs of Kaiser Permanente Washington (KP) and Seattle Children’s Hospital Odessa Brown Children’s Clinic (OBCC) approved protocols for pulling identifiable data and then sharing a limited, deidentified dataset. The study followed the Strengthening the Reporting of Observational Studies in Epidemiology (STROBE) reporting guideline.

### Study Sample

The sample was limited to children residing in 3 adjacent counties in Washington State: King (which includes but is not limited to Seattle), Pierce, and Snohomish. Children were aged between 2 and 18 years during the period from January 1, 2014, to December 31, 2019, and received primary care within 1 of 2 local health care systems (KP or OBCC). We limited the sample to children residing in an urban area or cluster (population >2500) as designated by the 2010 US Census. To avoid capturing unintentional weight loss, we excluded children who received a cancer diagnosis a year before or at any time during the observation period; we also excluded children who had undergone bariatric surgery at any time during their history with the health care system. We excluded observations that occurred within 9 months before and 3 months after a patient delivered a liveborn infant. We excluded children who either moved out of our study area or moved between Seattle and the nontaxed comparison areas. We limited the analysis to children who had at least 1 weight measurement in the medical record before and after the Seattle sweetened beverage tax was implemented. To account for potential data entry errors, we excluded children with extreme values for height-for-age *z* score (≤−5 or >4) or BMI *z* score (≤−5 or ≥10); we removed individuals with a very large change in BMI, BMI *z* score, or BMIp95, corresponding to the 1st and 99th percentiles of the distribution of change per year for each BMI metric. After applying exclusion criteria, we excluded children with missing covariates (eFigure 1 in Supplement 1).

### Exposure

The exposure of interest was the Seattle sweetened beverage tax (1.75 cents per ounce on sweetened beverages), defined by geography and timing of tax implementation (January 1, 2018). Addresses for children that were linked to each clinic visit were geocoded to determine whether they were inside or outside of Seattle. Due to different geocoding protocols for data from each health care system, we classified children as exposed to the tax for visits after January 1, 2018, if their geocoded address was inside the Seattle city limits for OBCC or in a US Census tract that was inside or touching the Seattle boundary for KP.

### Outcome

The primary outcome was each child’s BMIp95 (BMI expressed as a percentage of the 95th percentile) for an age- and sex-matched reference population according to the 2000 Centers for Disease Control and Prevention (CDC) growth charts. The BMIp95 is a newly recommended proxy of adiposity in children that is better at capturing change in BMI compared with a BMI *z* score, which has been shown to not accurately reflect change in BMI at the tails of the distribution.^18^ When comparing change in BMIp95, negative values suggest a reduction in child weight status (eAppendix 1 and eFigure 2 in Supplement 1). Weight and height were obtained from electronic health records. When weight was measured at a clinic visit but height was not measured on the same day, we imputed height for that child based on a random effects model of height growth over time (eAppendix 3 and eTable 1 in Supplement 1). In sensitivity analyses, we examined BMI *z* scores using 2022 CDC extended *z* scores and untransformed BMI.^18^

### Covariates

Both of our statistical models used statistical weighting techniques to create a well-matched comparison group that aimed to balance potential confounding variables. Weighting is an alternative form of statistically controlling for potential confounding variables, which generally involves up-weighting observations in the comparison group that look most similar to those in the treatment group and down-weighting observations that look dissimilar, with the goal of creating balance between the groups in the potentially confounding characteristics. In the primary (synthetic difference-in-differences [SDID]) model used in this study, the weighting was used to up-weight children with similar pretreatment trends in the outcome to children in Seattle, with the goal to achieve parallel trends. Both models estimated a within-person change and, therefore, controlled for all time-invariant, person-level factors.^19^ We additionally included 2 time-varying potential confounders: insurance status (to account for whether an individual changed insurance types from commercial to noncommercial from one point to the next) and an indicator for age group (to capture the general change in rate of BMI growth between ages 2-4 and 5-18 years).

For the secondary models, the variables used for weighting were at the individual and neighborhood levels. These variables included the following: age (continuous), sex (male or female), race and ethnicity (self-reported in categories and combined into the following options: American Indian or Alaska Native, Asian, Black or African American [hereinafter, Black], Hispanic, Native Hawaiian or Other Pacific Islander, White, other race or ethnicity [ie, any not listed], or multiple races or ethnicities), insurance type (commercial or other), pediatric medical complexity category (complex chronic conditions, noncomplex chronic conditions, or without chronic disease), US Census tract racial and ethnic composition (proportion of American Indian or Alaska Native, Asian, Black, Hispanic, Native Hawaiian or Other Pacific Islander, White, other race or ethnicity [ie, any not listed], or multiple races or ethnicities; continuous), proportion in the tract living in poverty (continuous), population density (people per square mile, continuous), proportion of the tract who have moved in the past year (continuous), proportion with a bachelor degree or higher (continuous), and interactions between individual race and ethnicity and population density and proportion with a bachelor degree or higher. Race and ethnicity are socially constructed categories that were included in this study because they are associated with both where people live (the exposure) and a multitude of other factors that influence BMI (the outcome). All tract-level variables were from the 2010 to 2014 American Community Survey 5-year estimates.

### Statistical Analysis

#### Primary Model: SDID Model

The SDID model combines aspects of synthetic control models^20^ and difference-in-differences models.^21^ The SDID method allows for individual-level repeated-measures data and for multiple treated units.^21,22,23^ It involves weighting to achieve balance on confounders; specifically, it reweights the sample to optimize matching children on parallel pretreatment trends in the outcome. In this study, the SDID method improved confidence that any differential change from before to after the policy was implemented was due to the tax. The models additionally used child fixed effects, which controlled for time-invariant, person-level confounders.^19^

The SDID model required a balanced sample; these models included only children who had at least 1 anthropometric measurement per year for every year of our observation period. For those who had more than 1 BMI measurement in a year, we used the mean of their BMIp95 measurements in each calendar year. Standard errors were estimated with a cluster bootstrap at the child level.^21,22^

Initial models indicated that observations from the earliest year in our observation period (2014) were not contributing substantially to the model weights. Therefore, we excluded 2014 from the SDID analysis and included children who had at least 1 measurement each year between 2015 and 2019. We additionally ran stratified versions of this model to assess whether the associations between the tax and BMIp95 were similar for subgroups by sex, age, race and ethnicity, insurance status, health care system, neighborhood poverty, pediatric medical complexity score, and baseline overweight. In sensitivity analyses, we examined BMI *z* scores and untransformed BMI.

#### Secondary Model: Fine Stratification Average Treatment Effect–Weighted, Within-Person Change Model

Because the synthetic control models were restricted to the balanced sample and because children who stayed in the same health care system and had at least 1 visit per year may be different from children who did not, we ran secondary models that were less restrictive and included any child who had at least 1 weight measurement before the tax and 1 after the tax was implemented. We used fine stratification-weighted, within-person change models, which are easier to interpret and allowed us a precise way to handle the fact that there was a varying amount of time that elapsed between measurements for each child. The fine stratification average treatment effect (FSATE) weights^24^ aimed to balance the sample characteristics between Seattle and the comparison area (eAppendix 2 in Supplement 1).

We used the BMIp95 value from the visit closest to, but not after, the last day before the tax (December 31, 2017) for the preperiod BMIp95. Then we subtracted this value from the BMIp95 measurement closest to, but not after, the last day in the posttax period for this study (January 1, 2020), and we divided this by the number of days between the measurement dates. We multiplied this value by 365.25 days in a year to create an annualized BMIp95 within-person change measure. We modeled annualized BMIp95 change as a function of Seattle residence or nonresidence, using FSATE weights to control for baseline confounders and adjusting for time-varying insurance type and age group.

All final statistical analyses were performed in Stata, version 18 (StataCorp LLC). Statistical significance was set to an α of .05 with 2-sided hypothesis tests. Analyses were conducted between August 5, 2022, and March 4, 2024.

## Results

The primary SDID model included 6313 children (3041 female [48%] and 3272 male [52%]) with at least 1 BMI measurement per year for all 5 years. Of these children, 1794 (28%) lived in Seattle and 4519 (72%) lived in the comparison area. The final sample in the FSATE-weighted change models included 22 779 unique children. Most children in the primary model were aged younger than 14 years at the first visit, consistent with the inclusion criteria, with 2383 (38%) aged 2 to 5 years (Table 1). The mean (SE) age of the 6313 children was 7.7 (0.6) years. In terms of race and ethnicity, 23 children (0.36%) were American Indian or Alaska Native, 789 (13%) were Asian, 631 (10%) were Black, 649 (10%) were Hispanic, 51 (0.81%) were Native Hawaiian or Other Pacific Islander, 3158 (50%) were White, 349 (5.5%) were of other race or ethnicity, and 663 (11%) were of multiple races or ethnicities.

**Table 1.** Characteristics of the Study Population^a^

Characteristic	SDID sample, unweighted			Full sample				
				Unweighted			FSATE weighted	
	Overall (N = 6313)	Seattle (n = 1794)	Comparison area (n = 4519)	Overall (N = 22 779)	Seattle (n = 6303)	Comparison area (n = 16 476)	Seattle (n = 6303)	Comparison area (n = 16 476)
Sex								
Female	48 (0.63)	49 (1.18)	48 (0.74)	49 (0.33)	49 (0.63)	48 (0.39)	50 (1.64)	47 (1.02)
Male	52 (0.63)	51 (1.18)	52 (0.74)	51 (0.33)	51 (0.63)	52 (0.39)	50 (1.64)	53 (1.02)
Age at first visit, y								
2-5	38 (0.61)	42 (1.17)	36 (0.71)	43 (0.33)	48 (0.63)	42 (0.38)	47 (1.63)	44 (1.03)
6-9	28 (0.56)	26 (1.04)	28 (0.67)	25 (0.29)	25 (0.54)	26 (0.34)	22 (1.30)	25 (0.86)
10-13	32 (0.59)	30 (1.08)	33 (0.70)	27 (0.29)	24 (0.54)	28 (0.35)	27 (1.48)	27 (0.89)
14-18	2.3 (0.19)	1.6 (0.30)	2.5 (0.23)	4.6 (0.14)	4.0 (0.25)	4.8 (0.17)	4.5 (0.71)	4.9 (0.47)
Self-reported race and ethnicity								
American Indian or Alaska Native	0.36 (0.08)	0.33 (0.14)	0.38 (0.09)	0.33 (0.04)	0.27 (0.07)	0.36 (0.05)	0.11 (0.04)	0.28 (0.04)
Asian	13 (0.42)	11 (0.73)	13 (0.50)	13 (0.22)	11 (0.39)	14 (0.27)	14 (1.2)	15 (0.86)
Black or African American	10 (0.38)	14 (0.83)	8.3 (0.41)	11 (0.21)	16 (0.46)	9.1 (0.22)	14 (1.1)	11 (0.74)
Hispanic	10 (0.38)	9.2 (0.68)	11 (0.46)	11 (0.20)	9.8 (0.37)	11 (0.24)	11 (1.0)	12 (0.73)
Native Hawaiian or Other Pacific Islander	0.81 (0.11)	0.45 (0.16)	0.95 (0.14)	1.2 (0.071)	0.73 (0.11)	1.3 (0.09)	2.1 (0.55)	1.2 (0.09)
White	50 (0.63)	50 (1.2)	50 (0.74)	48 (0.33)	47 (0.63)	48 (0.39)	43 (1.6)	44 (0.98)
Other^b^	5.5 (0.29)	6.5 (0.58)	5.2 (0.33)	6.0 (0.16)	6.8 (0.32)	5.7 (0.18)	6.2 (0.76)	6.5 (0.60)
Multiple	11 (0.39)	8.6 (0.66)	11 (0.47)	11 (0.20)	8.9 (0.36)	11 (0.25)	10 (1.0)	11 (0.69)
PMCA, maximum value								
1 (nonchronic)	54 (0.63)	58 (1.2)	52 (0.74)	65 (0.32)	69 (0.58)	64 (0.37)	66 (1.6)	65 (0.95)
2 (noncomplex chronic)	31 (0.58)	29 (1.1)	32 (0.69)	25 (0.29)	23 (0.53)	26 (0.34)	24 (1.4)	26 (0.89)
3 (complex chronic)	15 (0.45)	13 (0.78)	16 (0.55)	9.8 (0.20)	8.4 (0.35)	10 (0.24)	9.6 (0.99)	9.3 (0.50)
Insurance type								
Commercial	72 (0.57)	63 (1.1)	76 (0.64)	70 (0.30)	60 (0.62)	74 (0.34)	66 (1.51)	68 (1.13)
US Census tract race and ethnicity								
American Indian or Alaska Native	0.68 (0.02)	0.53 (0.02)	0.73 (0.02)	0.67 (0.01)	0.52 (0.01)	0.72 (0.01)	0.70 (0.03)	0.64 (0.02)
Asian	14 (0.14)	16 (0.33)	13 (0.14)	14 (0.07)	17 (0.17)	13 (0.08)	16 (0.26)	16 (0.38)
Black	6.8 (0.10)	9.8 (0.24)	5.7 (0.09)	6.9 (0.05)	11 (0.13)	5.5 (0.05)	7.9 (0.22)	6.7 (0.21)
Hispanic	9.4 (0.09)	7.6 (0.16)	10 (0.11)	9.5 (0.05)	7.7 (0.08)	10 (0.06)	13 (0.36)	9.8 (0.15)
Native Hawaiian or Other Pacific Islander	0.88 (0.02)	0.62 (0.03)	0.99 (0.03)	0.87 (0.01)	0.67 (0.02)	0.95 (0.01)	1.4 (0.07)	0.93 (0.03)
White	63 (0.24)	60 (0.56)	64 (0.24)	63 (0.12)	59 (0.30)	64 (0.13)	54 (0.56)	61 (0.41)
Other^b^	0.16 (0.005)	0.16 (0.010)	0.16 (0.006)	0.17 (0.003)	0.16 (0.005)	0.17 (0.003)	0.11 (0.009)	0.18 (0.009)
Multiple	5.5 (0.04)	5.4 (0.06)	5.5 (0.04)	5.4 (0.02)	5.4 (0.03)	5.4 (0.02)	6.1 (0.13)	5.3 (0.05)
US Census tract characteristic								
Density, mean (SE), people per square mile	5.3 × 10^3^ (45)	8.7 × 10^3^ (97)	3.9 × 10^3^ (33)	5.3 × 10^3^ (25)	8.9 × 10^3^ (58)	3.9 × 10^3^ (18)	5.8 × 10^3^ (1.0 × 10^2^)	5.5 × 10^3^ (1.8 × 10^2^)
Living in poverty	12 (0.10)	13 (0.21)	11 (0.11)	12 (0.05)	14 (0.12)	11 (0.06)	14 (0.31)	12 (0.18)
Moved in last year	17 (0.09)	18 (0.19)	16 (0.11)	17 (0.05)	18 (0.10)	16 (0.06)	18 (0.16)	19 (0.29)
College education or more	40 (0.25)	53 (0.45)	35 (0.26)	40 (0.13)	52 (0.24)	35 (0.14)	39 (0.62)	39 (0.57)

Abbreviations: FSATE, fine stratification average treatment effect; PMCA, pediatric medical complexity algorithm; SDID, synthetic difference-in-differences.

^a^ Unless otherwise indicated, values are listed as the mean percentage (SE).

^b^ Includes any race or ethnicity not captured in the categories listed.

When children in Seattle were compared with children in the comparison area in the primary SDID models (Table 1), the populations in Seattle were slightly younger, had a somewhat higher percentage of Black residents, had a lower percentage with commercial insurance, and had a lower proportion of children with complex, chronic conditions. US Census tracts of the Seattle sample had a lower proportion of Hispanic residents and a higher proportion of Black residents, higher population density, higher poverty levels, and a higher proportion in the tract with a college degree or higher (Table 1).

The FSATE weighting (Table 1) balanced well the moderate differences between the unweighted full sample for Seattle and the comparison area. Modest differences in child age and at the individual and tract levels remained.

The pretax mean BMIp95 in the Seattle sample was 83%, meaning that, on average, children’s BMI values were 83% of the BMI values at the 95th percentile using the CDC reference population (Table 2). All BMI metrics were higher in the comparison area. eTable 2 in Supplement 1 presents BMI metrics for a secondary sample.

**Table 2.** BMI Metrics for Seattle and the Comparison Area^a^

Metric	Overall		Before tax		After tax	
	Seattle	Comparison area	Seattle	Comparison area	Seattle	Comparison area
No. of observations	8970	22 595	5382	13 557	3588	9038
BMIp95	83 (0.15)	86 (0.11)	84 (0.18)	86 (0.13)	82 (0.25)	85 (0.19)
Pretax to posttax change in BMIp95	−3.8 (0.14)	−2.02 (0.09)	NA	NA	NA	NA
BMI	18 (0.04)	19 (0.03)	18 (0.05)	19 (0.04)	19 (0.07)	20 (0.06)
BMI *z* score^b^	0.26 (0.01)	0.44 (0.007)	0.25 (0.01)	0.40 (0.01)	0.27 (0.02)	0.48 (0.01)
Obesity prevalence, %	9.3 (0.31)	15 (0.24)	8.9 (0.39)	14 (0.30)	9.8 (0.50)	16 (0.39)

Abbreviations: BMI, body mass index (calculated as weight in kilograms divided by height in meters squared); BMIp95, BMI expressed as a percentage of the 95th percentile; FSATE, fine stratification average treatment effect; NA, not applicable; SDID, synthetic difference-in-differences.

^a^ Uses all the observations available during the study period from the 6313 children included in the SDID models. Values are presented as the mean (SE).

^b^ Calculated from the 2022 Centers for Disease Control and Prevention extended *z* score growth charts.

The SDID model created a sample with parallel trends in child BMIp95 during the pretax period between Seattle and the comparison area samples (Figure), as evidenced by the only very slightly increasing difference between the 2 groups in the pretax years. This difference increased substantially in the first year of the tax (2018) and grew in the second (2019) (Figure). These results suggest that the tax was associated with a greater decrease in BMIp95 for children in Seattle compared with the comparison area (SDID: −0.90 percentage points [95% CI, −1.2 to −0.60]; Table 3). Sensitivity analyses revealed that the findings were of similar direction and significance when using BMI *z* score and BMI as outcomes (Table 3 and eFigure 3 in Supplement 1). Findings from the FSATE-weighted change models of the association between the tax and BMIp95 were of somewhat larger magnitude compared with the SDID (β = −1.16 percentage points [95% CI, −1.91 to −0.41]; Table 3).

**Figure.**
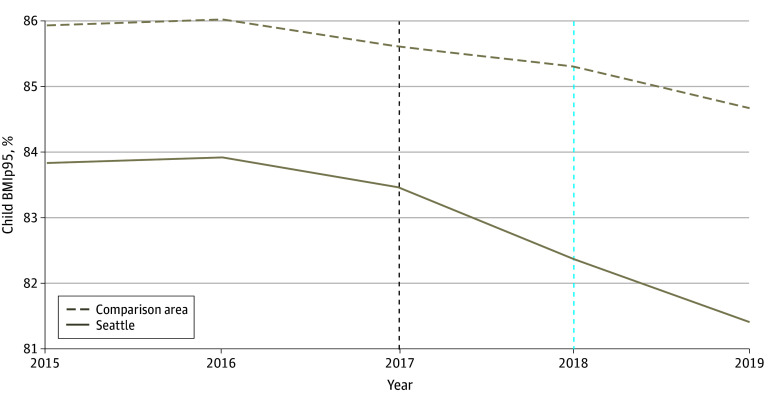
Estimated Trends and Synthetic Difference-in-Differences Estimates in BMIp95 Before and After Implementation of the Seattle Sweetened Beverage Tax The dashed black line indicates the end of the pretax period; the dashed blue line represents the first year of outcomes after tax implementation, encompassing measurements between January 1 and December 31, 2018. BMIp95 indicates body mass index (calculated as weight in kilograms divided by height in meters squared) expressed as a percentage of the 95th percentile.

**Table 3.** Association Between the Seattle Sweetened Beverage Tax and BMIp95 and Sensitivity Analyses With Extended BMI *z* Scores and BMI^a^

Metric	Difference-in-differences estimate		No. of children^b^
	Coefficient (95% CI)	*P* value	
BMIp95			
SDID	−0.90 (−1.2 to −0.60)	<.001	6313
Annualized change	−1.16 (−1.91 to −0.41)	.004	22 779
BMI *z* score (extended) sensitivity analysis			
SDID	−0.05 (−0.07 to −0.03)	<.001	6313
Annualized change	−0.09 (−0.15 to −0.02)	<.006	22 779
BMI (untransformed) sensitivity analysis			
SDID	−0.22 (−0.30 to −0.14)	<.001	6313
Annualized change	−0.36 (−0.59 to −0.13)	.002	22 779

Abbreviations: BMI, body mass index (calculated as weight in kilograms divided by height in meters squared); BMIp95, BMI expressed as a percentage of the 95th percentile; FSATE, fine stratification average treatment effect; SDID, synthetic difference-in-differences.

^a^ Results are presented for SDID (primary model) and FSATE-weighted first change models (secondary model).

^b^ The SDID models use a balanced panel of 5 observations from each unique child or 31 565 total observations.

The stratified SDID models suggested that for many demographic groups, the direction of the estimate of the association between the tax and BMI was negative; that is, children in Seattle gained less BMIp95 than children in the comparison area from before to after implementation of the tax (Table 4). This finding was statistically significant for males and females, for younger and older children, both insurance types, for the KP health care system, for some racial and ethnic populations (ie, Black, White, or other race), for high-poverty and low-poverty neighborhoods, and for all levels of pediatric medical complexity. The association was large and negative among patients with baseline overweight.

**Table 4.** Associations Between the Seattle Sweetened Beverage Tax and BMIp95 Using Population Group-Stratified SDID Models

Characteristic	Coefficient (95% CI)	*P* value	No. of observations
Sex			
Male	−0.85 (−1.3 to −0.42)	<.001	16 360
Female	−0.93 (−1.4 to −0.50)	<.001	15 205
Age, y			
2-4	−0.78 (−1.2 to −0.38)	<.001	12 360
5-18	−0.87 (−1.3 to −0.46)	<.001	19 205
Health care system			
KP only	−0.97 (−1.3 to −0.62)	<.001	28 715
OBCC only	−0.81 (−2.0 to 0.34)	.17	2850
Self-reported race and ethnicity			
American Indian or Alaska Native	−2.8 (−13 to 7.1)	.58	115
Asian	0.12 (−0.72 to 0.96)	.78	3945
Black	−1.4 (−2.4 to −0.46)	.004	3155
Hispanic	−0.49 (−1.5 to −0.48)	.32	3245
Native Hawaiian or Other Pacific Islander	3.5 (−0.31 to 7.4)	.07	255
White	−1.2 (−1.6 to −0.81)	<.001	15 790
Other^a^	−1.5 (−2.8 to −0.24)	.02	1745
Multiple	−0.51 (−1.4 to 0.33)	.23	3315
Insurance type			
Commercial	−0.78 (−1.2 to −0.40)	<.001	23 140
Other than commercial	−1.3 (−1.8 to −0.77)	<.001	8425
Neighborhood poverty			
High	−1.1 (−1.7 to −0.39)	.002	6015
Low	−0.87 (−1.2 to −0.55)	<.001	25 550
PMCA maximum value			
1	−0.80 (−1.2 to −0.42)	<.001	16 905
2	−0.85 (−1.4 to −0.31)	.002	9880
3	−1.2 (−2.3 to −0.11)	.03	4780
Baseline overweight status			
Overweight or obesity	−1.7 (−2.6 to −0.85)	<.001	8110
No overweight or obesity	−0.64 (−0.94 to −0.34)	<.001	23 455

Abbreviations: BMI, body mass index (calculated as weight in kilograms divided by height in meters squared); BMIp95, BMI expressed as a percentage of the 95th percentile; KP, Kaiser Permanente Washington; OBCC, Seattle Children’s Hospital Odessa Brown Children’s Clinic; PMCA, pediatric medical complexity algorithm; SDID, synthetic difference-in-differences.

^a^ Includes any race or ethnicity not captured in the categories listed.

## Discussion

In this study, the Seattle sweetened beverage tax (1.75 cents per ounce on sweetened beverages) was associated with a statistically significant reduction in children’s BMIp95. Additionally, statistically significant reductions in BMIp95 were observed for many subgroups, and decreases in alternative BMI outcomes (BMI *z* score [extended] and BMI) were also observed. These findings are consistent with our expectations given the modest scale of the tax and the complex social and behavioral mechanisms hypothesized to underlie current obesity trends and with outcomes suggested by modeling studies.^25^

The SDID model derived a weighted sample of comparison area participants who had similar pretax trends in the outcome as those in Seattle. This approach increased our confidence that any association could be attributed to the tax rather than to preexisting, unobserved factors. We selected it after our prespecified approaches inadequately controlled for factors creating different BMIp95 trajectories in the pretax period, whereas the SDID model eliminated those pretax differences.^26^ Thus, it was important to choose a model that prioritized matching on prepolicy outcomes to up-weight children in the comparison area who were experiencing the same longitudinal trends in BMIp95 before tax implementation as those in Seattle.

Our findings are consistent with those of previous studies. Flynn^17^ investigated the combined areas of Philadelphia, San Francisco, and Oakland and reported an average decrease in children’s BMI from before to after beverage taxes were implemented. Additionally, a study from Mexico^16^ reported an association between Mexico’s tax and decreased BMI among girls. Whereas these studies had repeated cross-sectional designs, our study used longitudinal data from the same children over time, used measured height and weight, and implemented methods to robustly control for pretax differences in trends; thus, our study builds on and adds rigor to the evidence. Our findings are also consistent with findings of net reductions of 22% of volume sold of taxed beverage and net reductions in added sugar purchased from beverages in Seattle and Philadelphia, which, if uncompensated, would be expected to result in improved BMI.^11,27,28^

Our results are less consistent with findings from our previous longitudinal cohort study of taxed beverage consumption among lower-income children in Seattle and the nearby nontaxed comparison area.^29^ In that study, we found no greater reduction in reported consumption for Seattle children vs those in the comparison area.^29^ However, dietary consumption is difficult to measure,^30^ and multiple studies often have no evidence of decreases in self-reported consumption in places where substantial decreases in sweetened beverage purchasing were seen.

### Limitations

This study has some limitations. Data were not available for sweetened beverage consumption, so our sample was not limited to these consumers. Thus, the associations are diluted relative to what would be expected in a cohort restricted to those who consumed any sweetened beverages at baseline. Other limitations include our use of medical record data with limited information about individual household economic status or other characteristics. However, we used child-level fixed effects or differencing, which compared children to themselves over time and controlled for all time-invariant child-level confounders. The SDID model required that all children had the same number of outcome measurements. The trade-off for the smaller sample and lower generalizability was an internally valid estimate of the implications of the Seattle sweetened beverage tax on children’s BMIp95. The fuller sample model demonstrated a similar pattern of results. The BMIp95 is cumbersome to describe and interpret. We cannot rule out that an unknown confounder might account for the differences between the pretax and posttax periods; to explain the difference, this unknown confounder would have to be similarly timed with the tax.

## Conclusions

The results of this cohort study suggest that the Seattle sweetened beverage tax was associated with a decrease in BMIp95 among children, as evidenced by larger decreases from before to after implementation of the tax among children living in Seattle vs the nearby nontaxed comparison area. Taken together with existing studies in the US, these results suggest that sweetened beverage taxes may be an effective policy for improving children’s BMI. Future research should test this association using longitudinal data in other US cities with sweetened beverage taxes.

## Notes

Supplement 1. **eFigure 1.** Diagram of Study Sample Inclusion Criteria and Sample Size **eAppendix 1.** Body Mass Index (BMI) as a Percent of the Value at the 95th Percentile (BMIp95) **eFigure 2.** Chart Depicting the Percent of the 95th Percentile of Body Mass Index by Sex—An Alternative Metric for Measuring BMI **eAppendix 2.** Weighting to Balance Seattle and Comparison Area for Use in Within-Person Change Models **eAppendix 3.** Height Imputation Details **eTable 1.** Comparisons of the Association Between the Seattle Sweetened Beverage Tax and BMIp95 **eTable 2.** BMI Metrics for Seattle and Comparison Area
eReferences
**eFigure 3.** Synthetic Difference-in-Differences Plots for BMI *z* Score (Extended) and BMI Untransformed

Supplement 2.
Data Sharing Statement
