# Supplementary material for: Sweetened Beverage Tax Implementation and Change in Body Mass Index Among Children in Seattle
Source: JAMA Netw Open. 2024 May 29;7(5):e2413644. doi: 10.1001/jamanetworkopen.2024.13644 (PMC11137635; doi:10.1001/jamanetworkopen.2024.13644)
Supplement: Supplement 1. — eFigure 1. Diagram of Study Sample Inclusion Criteria and Sample Size eAppendix 1. Body Mass Index (BMI) as a Percent of the Value at the 95th Percentile (BMIp95) eFigure 2. Chart Depicting the Percent of the 95th Percentile of Body Mass Index by Sex—An Alternative Metric for Measuring BMI eAppendix 2. Weighting to Balance Seattle and Comparison Area for Use in Within-Person Change Models eAppendix 3. Height Imputation Details eTable 1. Comparisons of the Association Between the Seattle Sweetened Beverage Tax and BMIp95 eTable 2. BMI Metrics for Seattle and Comparison Area eReferences eFigure 3. Synthetic Difference-in-Differences Plots for BMI z Score (Extended) and BMI Untransformed [file jamanetwopen-e2413644-s001.pdf]

Supplementary Online Content

Jones-Smith JC, Knox M, Chakrabarti S, et al. Sweetened beverage tax implementation and change in body mass index among children in Seattle. *JAMA Netw Open*. 2024;7(5):e2413644. doi:10.1001/jamanetworkopen.2024.13644

eFigure 1. Diagram of Study Sample Inclusion Criteria and Sample Size

eAppendix 1. Body Mass Index (BMI) as a Percent of the Value at the 95th Percentile (BMIp95)

eFigure 2. Chart Depicting the Percent of the 95th Percentile of Body Mass Index by Sex—An Alternative Metric for Measuring BMI

eAppendix 2. Weighting to Balance Seattle and Comparison Area for Use in Within-Person Change Models

eAppendix 3. Height Imputation Details

eTable 1. Comparisons of the Association Between the Seattle Sweetened Beverage Tax and BMIp95

eTable 2. BMI Metrics for Seattle and Comparison Area

eReferences

eFigure 3. Synthetic Difference-in-Differences Plots for BMI *z* Score (Extended) and BMI Untransformed

This supplementary material has been provided by the authors to give readers additional information about their work.

© 2024 Jones-Smith JC et al. *JAMA Network Open.*

eFigure 1. Diagram of Study Sample Inclusion Criteria and Sample Size

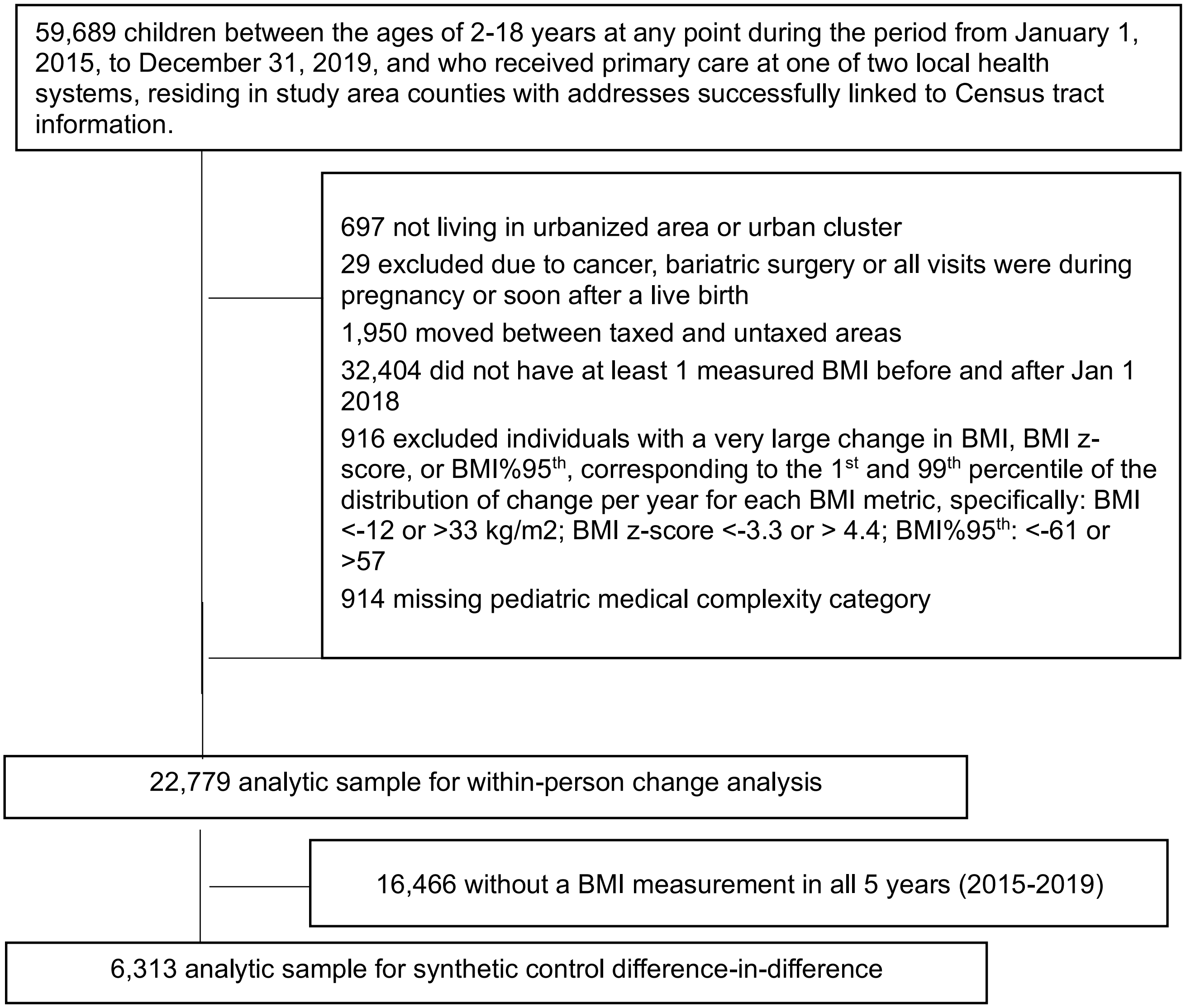

© 2024 Jones-Smith JC et al. *JAMA Network Open.*

eAppendix 1. Body Mass Index (BMI) as a Percent of the Value at the 95th Percentile (BMIp95)

Our outcome was each child's BMI expressed as a percentage of the BMI value of the 95th percentile of BMI for an age- and sex-matched reference population of the CDC/NCHS 2000 Growth Charts (henceforth abbreviated BMI%95th), which is a newly recommended measure[1] of BMI that is better at capturing change in BMI among populations in which overweight and obesity are prevalent, such as among U.S. children. For readers more familiar with BMI growth curves, these images below from the CDC show the BMI%95th values overlayed onto the CDC BMI for age and sex growth curves. A value of 80% is above the median, but below the 95th percentile.[1] When comparing change in the BMI%95th, lower numbers imply a lower weight status and negative values suggest a reduction in child weight status. For example, for a child who had a BMI of 18 kg/m^2 and the reference BMI of the 95th percentile for age and sex was 17 kg/m^2 at their first visit, their BMI%95th would be (18/17)*100= 105.9% of the BMI value at that 95th percentile of the distribution. If at their second visit, their BMI was 17.5 kg/m^2 and the referent BMI for their age was now 18, then their BMI%95th would be (17.5/18)*100 = 97.2%. Change over time would be second visit BMI%95th (97.2) – first visit BMI%95th (105.9) = -8.7 percentage point (pp) change.

© 2024 Jones-Smith JC et al. *JAMA Network Open.*

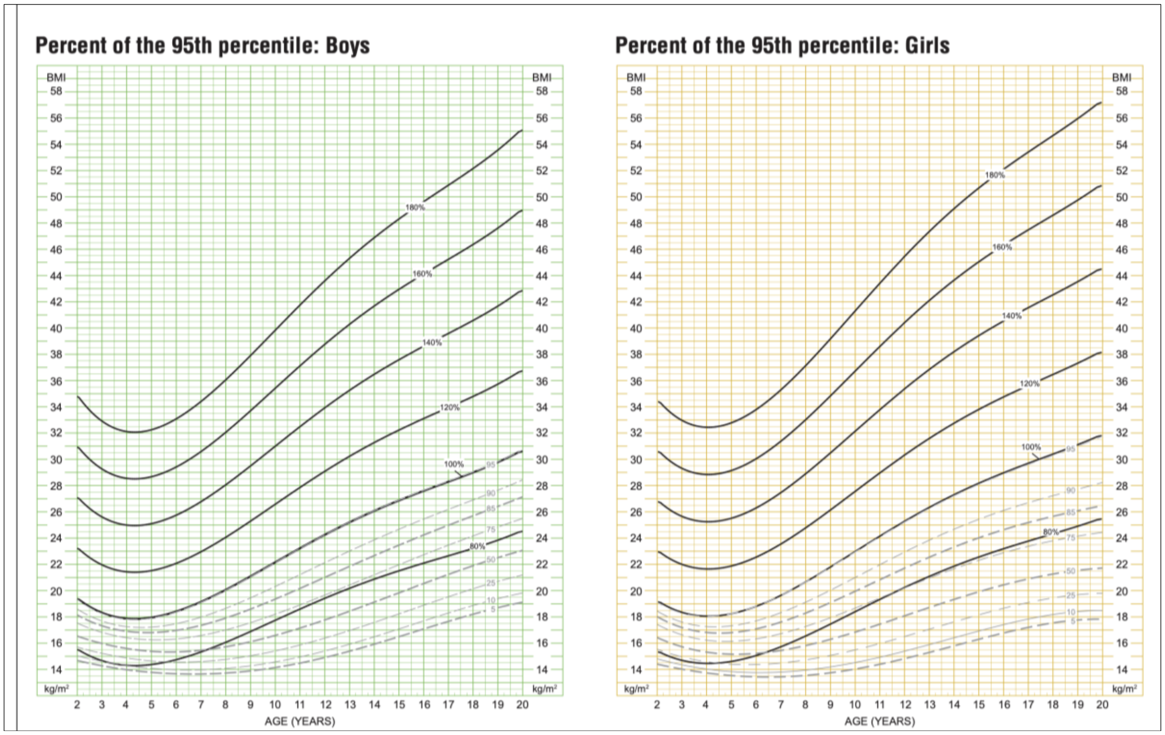

eFigure 2. Chart Depicting the Percent of the 95th Percentile of Body Mass Index by Sex—An Alternative Metric for Measuring BMI

© 2024 Jones-Smith JC et al. *JAMA Network Open.*

eAppendix 2. Weighting to Balance Seattle and Comparison Area for Use in Within-Person Change Models

We used fine stratification propensity score weighting to achieve balance on measured confounders for people living in Seattle and the comparison area (people living outside of Seattle in urban areas of the three surrounding counties). In the propensity score model, we included variables we hypothesized to be associated with both the outcome and the probability of living in Seattle, which were the time-invariant variables described in the Covariates section of the manuscript. Balancing on these factors is a way of eliminating their effect on the outcome so that we can isolate the effect of the tax on BMI.

We used a weighting method called fine stratification average treatment effect (FSATE) weighting since the distribution of the propensity scores was substantially skewed and the treated group was smaller than comparison group.[2] The fine stratification weights, which lower the influence of any individual having a weight close to 0 or 1, are created by first estimating the propensity or probability of being in the treatment group (live in a census tract in Seattle) as a function of the covariates, using a logistic regression model. We then stratify the propensity scores into the following bins separately within Seattle and within the comparison area: [[0, 0.1), [0.1, 0.2), …, [0.9, 1]]. Every individual within a specific stratum receives the same weight based on whether they live in Seattle or the comparison area, which is the ratio of the proportion of all individuals whose propensity scores fall in that strata to the proportion of individuals in Seattle or the comparison area, respectively, in that strata. FSATE weights aim to balance the sample characteristics between Seattle ("treatment" group) and the comparison area (outside of Seattle) and resultingly make the weighted pseudo-population sample look more like the overall averages of the sample on covariates used to construct weights. The ATE when using the FSATE weights represents the average effect that would be expected if everyone in the population were exposed to the treatment (i.e. the tax).

eAppendix 3. Height Imputation Details

We imputed height when height was missing but weight was not missing for a visit. We did so using a random effects model with a random intercept for child and random slope for child age. The distribution of the imputed values was very well aligned with the distribution of non-imputed values. Height was imputed for 28% of the observations in the within-person change models and for 34% in the synthetic DID models. While the imputation likely introduces noise to the data, this error would be expected to be non-differential by treatment status (i.e. Seattle versus Comparison Area), and thus would be expected to attenuate any associations that exist. The mean difference between imputed and actual height for children who have both in the synthetic DID sample is 0.12 cm in Seattle and 0.13 in the comparison area. A t-test for the null hypothesis that these are not different gives the p-value of 0.60. For the within-person change models, average difference in Seattle is 0.56 cm overall, 0.63 in Seattle and 0.52 in the comparison area and the p-value for the t-test for the null hypothesis that these are not different is 0.51. Both of these t-tests support the idea that the error is non-differential by treatment status.

For thoroughness, we re-ran the within-person change models using only observations with non-imputed height. The results are consistent with the main results and are indeed of larger magnitude despite a substantially reduced sample size (shown in table below)

For the synthetic control models, because these models require a balanced panel of observations, limiting the analysis sample to children who have height measured during all five years results in a very large reduction in sample. Instead, to test the sensitivity of the results to imputation, we run the synthetic control on a sample of three years with 2 pre-policy years (2016 & 2017) and 1 post policy year (2018). The results from this shorter time frame are also consistent with the primary analysis in terms of general size of the point estimate and statistical significance despite a substantial reduction in sample size.

© 2024 Jones-Smith JC et al. *JAMA Network Open.*

eTable 1. Comparisons of the Association Between the Seattle Sweetened Beverage Tax and BMIp95

Sensitivity analysis limiting to observations for which height was not imputed; annualized change model and synthetic difference-in-differences (primary estimates also shown for easy comparisons)

	Difference-in-Difference Estimate		N[a]
	Coefficient (SE)	p-value	
Annualized change in BMI%95th	-1.16 (0.38)	0.002	22,779
Annualized change in BMI%95th, for those with non-imputed height only	-1.41 (0.44)	0.002	16,360
Synthetic difference-in-difference of BMI%95th (estimates in main paper)	-0.9 (0.16)	<0.001	6,313
Synthetic difference-in-difference of BMIp95, for those with all height measurements, 2016, 2017, 2018	-0.85 (0.24)	<0.001	2,135
Synthetic difference-in-difference of BMIp95, for those including those with imputed height measurements, 2016, 2017, 2018 only	-0.76 (0.14)	<0.001	6,313

[a] N is the number of unique children. The new synthetic difference-in-difference models use a balanced panel of 3 observations from each unique child.

© 2024 Jones-Smith JC et al. *JAMA Network Open.*

eTable 2. BMI Metrics for Seattle and Comparison Area

	Seattle, Full Sample, unweighted	Comparison, Full Sample, Unweighted	Seattle, Full Sample, FSATE-weighted	Comparison, Full Sample, FSATE-weighted
	Mean (SE)			
	Overall			
Observations	***56,737***	***152,388***	***56,737***	***152,388***
BMI%95th	84 (0.062)	86 (0.043)	86 (0.17)	86 (0.1)
BMI	18 (0.018)	19 (0.013)	19 (0.053)	19 (0.027)
BMI z-score	0.27 (0.0046)	0.44 (0.0029)	0.39 (0.012)	0.43 (0.0068)
Avg within individual Change in BMI%95th	-2.4 (0.050)	-1.2 (0.031)	-1.4 (0.12)	-0.13 (0.086)
Avg within individual Change in BMI z-score	-0.0037 (0.0038)	0.045 (0.0023)	0.035 (0.0091)	0.12 (0.0059)
Avg within individual Change in BMI	1.5 (0.015)	1.8 (0.0094)	1.4 (0.033)	2.1 (0.037)
Obesity Prevalence, (%)	10 (0.13)	15 (0.092)	13 (0.39)	15 (0.23)
	Pre-tax			
Observations	***36,387***	***97,847***	***36,387***	***97,847***
BMI%95th	84 (0.075)	86 (0.0451	86 (0.21)	86 (0.11)
BMI	18 (0.021)	19 (0.015)	19 (0.064)	19 (0.028)
BMI z-score	0.25 (0.0058)	0.40 (0.0036)	0.39 (0.015)	0.38 (0.0083)

© 2024 Jones-Smith JC et al. *JAMA Network Open.*

Obesity Prevalence, (%)	9.9 (0.16)	15 (0.11)	13 (0.48)	14 (0.26)
	**Post-tax**			
Observations	***20,350***	***54,541***	***20,350***	***54,541***
BMI%95th	83 (0.11)	86 (0.077)	85 (0.30)	87 (0.19)
BMI	19 (0.032)	20 (0.023)	20 (0.093)	20 (0.052)
BMI z-score	0.30 (0.0076)	0.50 (0.0048)	0.39 (0.019)	0.51 (0.012)
Obesity Prevalence, (%)	11 (0.22)	16 (0.16)	13 (0.64)	17 (0.44)

eReferences

1. Hales CM, Freedman D, Akinbami L, Wei R, Ogden C. *Evaluation of Alternative Body Mass Index (BMI) Metrics to Monitor Weight Status in Children and Adolescents with Extremely High BMI Using CDC BMI-for-Age Growth Charts*. National Center for Health Statistics (U.S.); 2022. doi:10.15620/cdc:121711

2. Desai RJ, Franklin JM. Alternative approaches for confounding adjustment in observational studies using weighting based on the propensity score: a primer for practitioners. *BMJ*. Published online October 23, 2019:l5657. doi:10.1136/bmj.l5657

© 2024 Jones-Smith JC et al. *JAMA Network Open.*

eFigure 3. Synthetic Difference-in-Differences Plots for BMI *z* Score (Extended) and BMI Untransformed

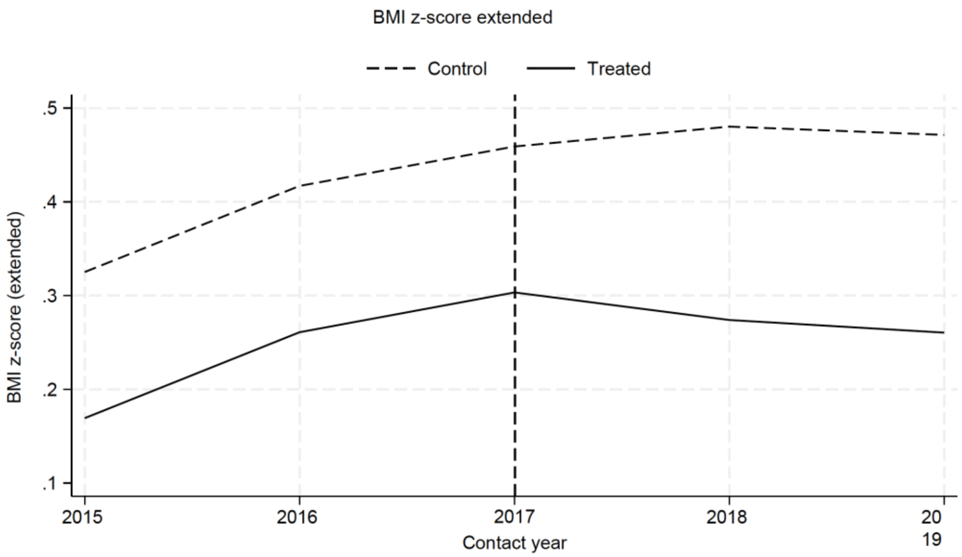

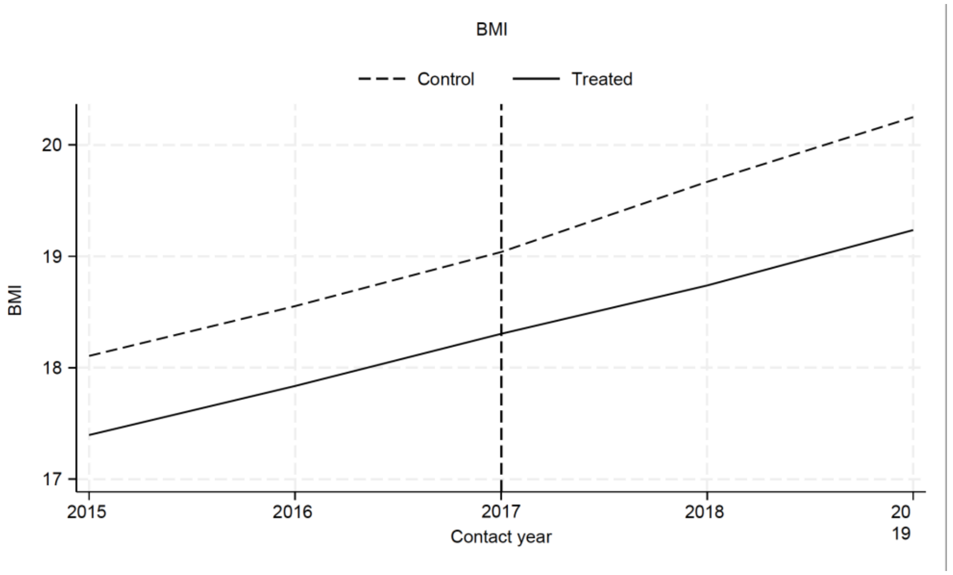

© 2024 Jones-Smith JC et al. *JAMA Network Open.*
