# Supplementary material for: Sweetened Beverage Tax Implementation and Change in Body Mass Index Among Children in Seattle
Source: JAMA Netw Open. 2024 May 29;7(5):e2413644. doi: 10.1001/jamanetworkopen.2024.13644 (PMC11137635; doi:10.1001/jamanetworkopen.2024.13644)
Supplement: Supplement 2. — Data Sharing Statement [file jamanetwopen-e2413644-s002.pdf]

Data Sharing Statement

Jones-Smith. Sweetened Beverage Tax Implementation and Change in Body Mass Index Among Children in Seattle. *JAMA Netw Open*. Published May 29, 2024. doi:10.1001/jamanetworkopen.2024.13644

Data
Data available: No

Additional Information
Explanation for why data not available: Our data sharing agreement with the health systems that provided the patient data do not allow for sharing further
